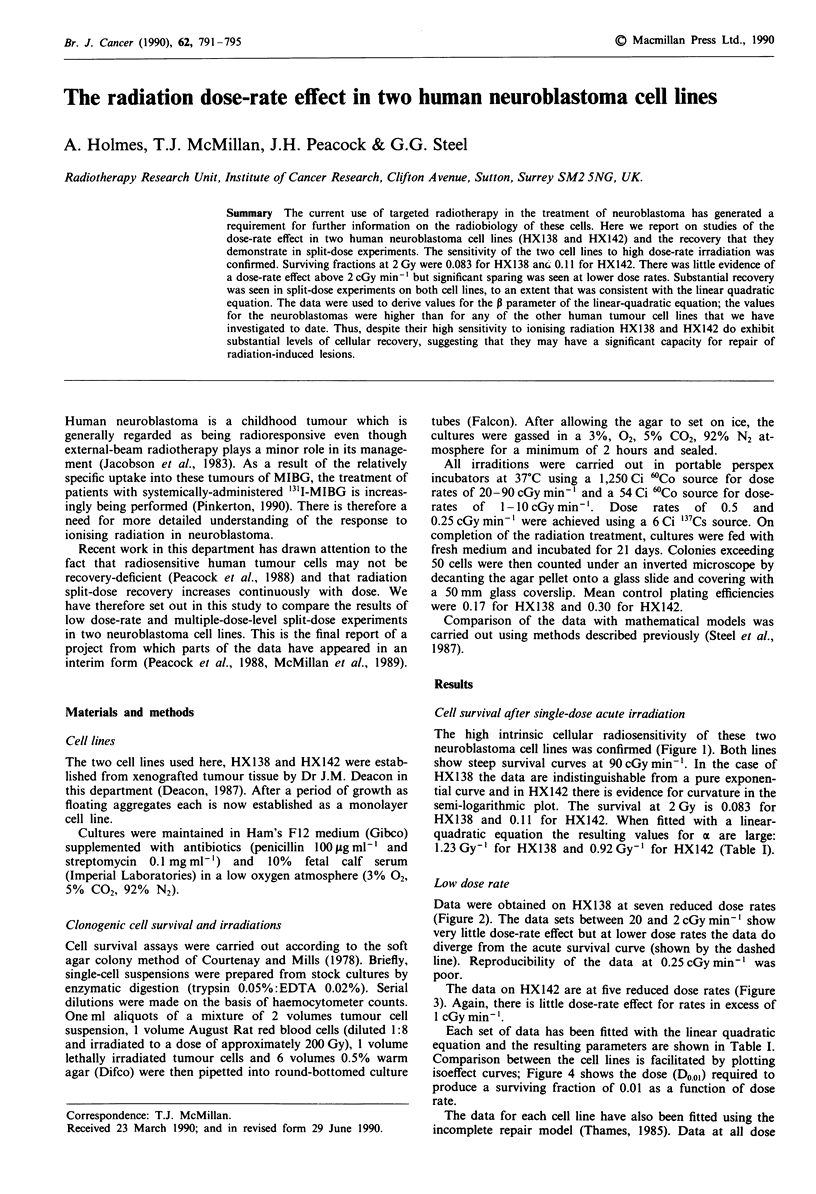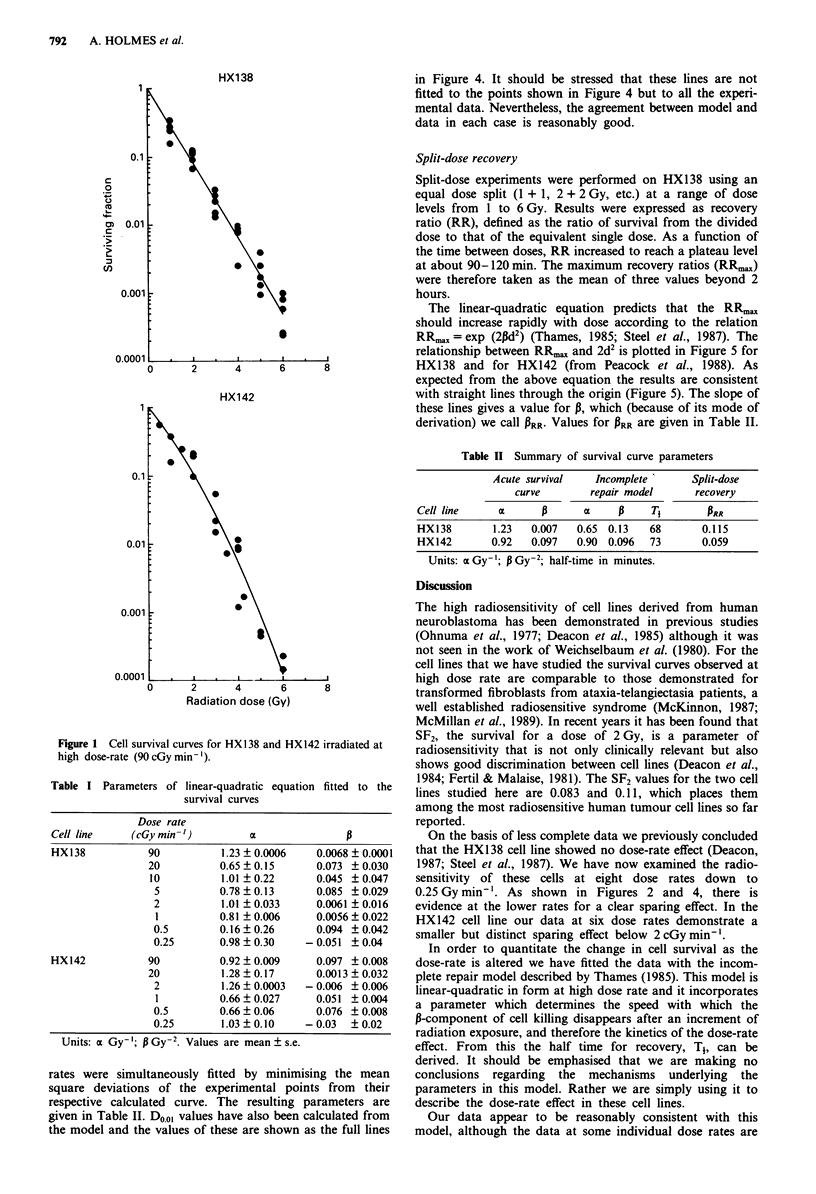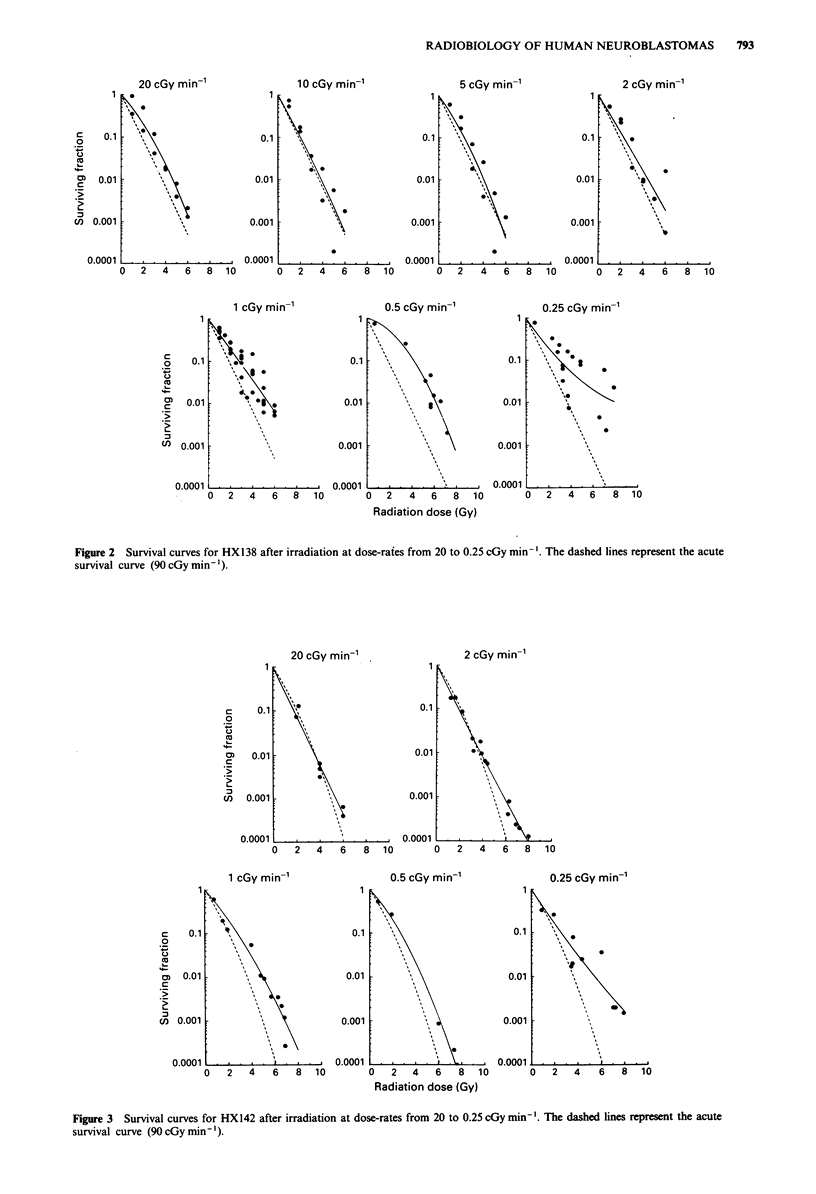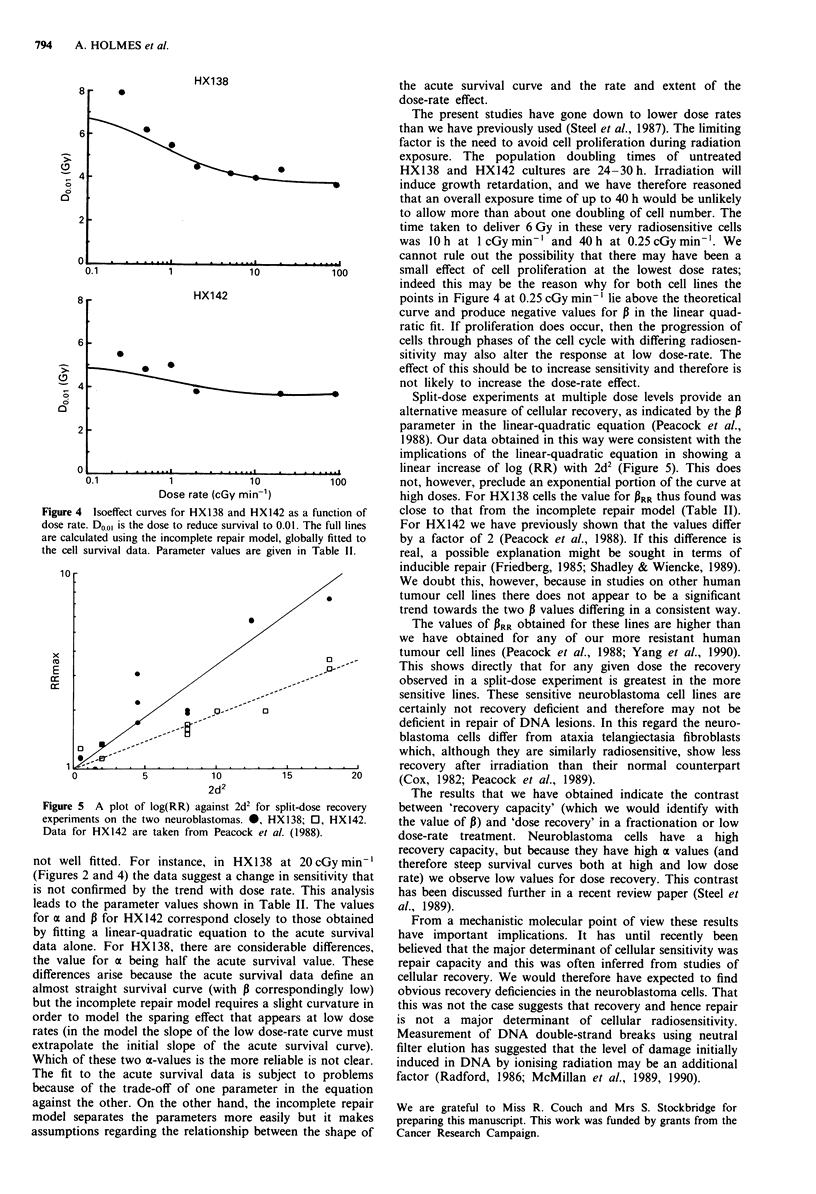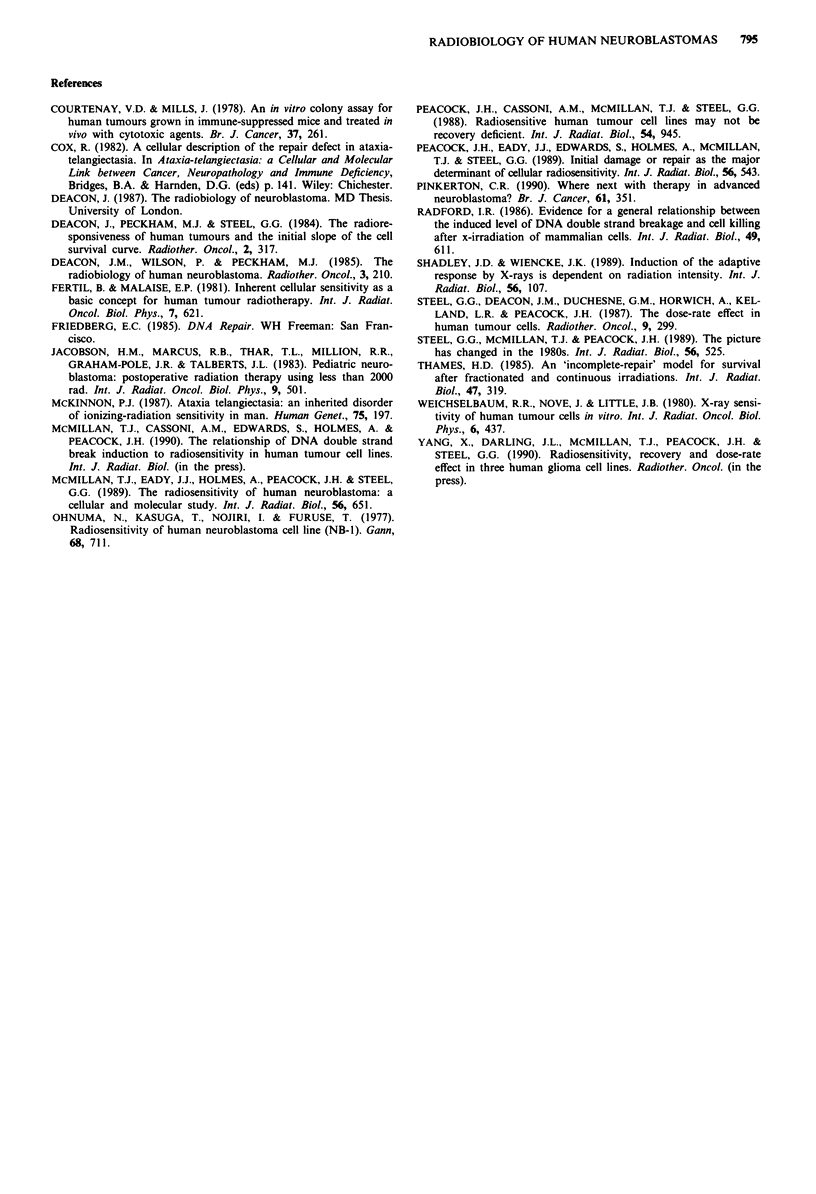# The radiation dose-rate effect in two human neuroblastoma cell lines.

**DOI:** 10.1038/bjc.1990.381

**Published:** 1990-11

**Authors:** A. Holmes, T. J. McMillan, J. H. Peacock, G. G. Steel

**Affiliations:** Radiotherapy Research Unit, Institute of Cancer Research, Sutton, Surrey, UK.

## Abstract

The current use of targeted radiotherapy in the treatment of neuroblastoma has generated a requirement for further information on the radiobiology of these cells. Here we report on studies of the dose-rate effect in two human neuroblastoma cell lines (HX138 and HX142) and the recovery that they demonstrate in split-dose experiments. The sensitivity of the two cell lines to high dose-rate irradiation was confirmed. Surviving fractions at 2 Gy were 0.083 for HX138 and 0.11 for HX142. There was little evidence of a dose-rate effect above 2 cGy min-1 but significant sparing was seen at lower dose rates. Substantial recovery was seen in split-dose experiments on both cell lines, to an extent that was consistent with the linear quadratic equation. The data were used to derive values for the beta parameter of the linear-quadratic equation; the values for the neuroblastomas were higher than for any of the other human tumour cell lines that we have investigated to date. Thus, despite their high sensitivity to ionising radiation HX138 and HX142 do exhibit substantial levels of cellular recovery, suggesting that they may have a significant capacity for repair of radiation-induced lesions.


Br. J. Cancer (1990), 62, 791-795

© Macmillan Press Ltd., 1990

### The radiation dose-rate effect in two human neuroblastoma cell lines

A. Holmes, T.J. McMillan, J.H. Peacock & G.G. Steel

*Radiotherapy Research Unit, Institute of Cancer Research, Clifton Avenue, Sutton, Surrey SM2 5NG, UK.*

**Summary** The current use of targeted radiotherapy in the treatment of neuroblastoma has generated a requirement for further information on the radiobiology of these cells. Here we report on studies of the dose-rate effect in two human neuroblastoma cell lines (HX138 and HX142) and the recovery that they demonstrate in split-dose experiments. The sensitivity of the two cell lines to high dose-rate irradiation was confirmed. Surviving fractions at 2 Gy were 0.083 for HX138 and 0.11 for HX142. There was little evidence of a dose-rate effect above 2 cGy $min^{-1}$ but significant sparing was seen at lower dose rates. Substantial recovery was seen in split-dose experiments on both cell lines, to an extent that was consistent with the linear quadratic equation. The data were used to derive values for the $\beta$ parameter of the linear-quadratic equation; the values for the neuroblastomas were higher than for any of the other human tumour cell lines that we have investigated to date. Thus, despite their high sensitivity to ionising radiation HX138 and HX142 do exhibit substantial levels of cellular recovery, suggesting that they may have a significant capacity for repair of radiation-induced lesions.

Human neuroblastoma is a childhood tumour which is generally regarded as being radioresponsive even though external-beam radiotherapy plays a minor role in its management (Jacobson *et al.*, 1983). As a result of the relatively specific uptake into these tumours of MIBG, the treatment of patients with systemically-administered $^{131}$I-MIBG is increasingly being performed (Pinkerton, 1990). There is therefore a need for more detailed understanding of the response to ionising radiation in neuroblastoma.

Recent work in this department has drawn attention to the fact that radiosensitive human tumour cells may not be recovery-deficient (Peacock *et al.*, 1988) and that radiation split-dose recovery increases continuously with dose. We have therefore set out in this study to compare the results of low dose-rate and multiple-dose-level split-dose experiments in two neuroblastoma cell lines. This is the final report of a project from which parts of the data have appeared in an interim form (Peacock *et al.*, 1988, McMillan *et al.*, 1989).

### Materials and methods

### *Cell lines*

The two cell lines used here, HX138 and HX142 were established from xenografted tumour tissue by Dr J.M. Deacon in this department (Deacon, 1987). After a period of growth as floating aggregates each is now established as a monolayer cell line.

Cultures were maintained in Ham's F12 medium (Gibco) supplemented with antibiotics (penicillin 100 $\mu$g $ml^{-1}$ and streptomycin 0.1 mg $ml^{-1}$) and 10% fetal calf serum (Imperial Laboratories) in a low oxygen atmosphere (3% $O_2$, 5% $CO_2$, 92% $N_2$).

### *Clonogenic cell survival and irradiations*

Cell survival assays were carried out according to the soft agar colony method of Courtenay and Mills (1978). Briefly, single-cell suspensions were prepared from stock cultures by enzymatic digestion (trypsin 0.05%:EDTA 0.02%). Serial dilutions were made on the basis of haemocytometer counts. One ml aliquots of a mixture of 2 volumes tumour cell suspension, 1 volume August Rat red blood cells (diluted 1:8 and irradiated to a dose of approximately 200 Gy), 1 volume lethally irradiated tumour cells and 6 volumes 0.5% warm agar (Difco) were then pipetted into round-bottomed culture tubes (Falcon). After allowing the agar to set on ice, the cultures were gassed in a 3%, $O_2$, 5% $CO_2$, 92% $N_2$ atmosphere for a minimum of 2 hours and sealed.

All irraditions were carried out in portable perspex incubators at 37°C using a 1,250 Ci $^{60}$Co source for dose rates of 20–90 cGy $min^{-1}$ and a 54 Ci $^{60}$Co source for dose-rates of 1–10 cGy $min^{-1}$. Dose rates of 0.5 and 0.25 cGy $min^{-1}$ were achieved using a 6 Ci $^{137}$Cs source. On completion of the radiation treatment, cultures were fed with fresh medium and incubated for 21 days. Colonies exceeding 50 cells were then counted under an inverted microscope by decanting the agar pellet onto a glass slide and covering with a 50 mm glass coverslip. Mean control plating efficiencies were 0.17 for HX138 and 0.30 for HX142.

Comparison of the data with mathematical models was carried out using methods described previously (Steel *et al.*, 1987).

### Results

### *Cell survival after single-dose acute irradiation*

The high intrinsic cellular radiosensitivity of these two neuroblastoma cell lines was confirmed (Figure 1). Both lines show steep survival curves at 90 cGy $min^{-1}$. In the case of HX138 the data are indistinguishable from a pure exponential curve and in HX142 there is evidence for curvature in the semi-logarithmic plot. The survival at 2 Gy is 0.083 for HX138 and 0.11 for HX142. When fitted with a linear-quadratic equation the resulting values for $\alpha$ are large: 1.23 $Gy^{-1}$ for HX138 and 0.92 $Gy^{-1}$ for HX142 (Table I).

### *Low dose rate*

Data were obtained on HX138 at seven reduced dose rates (Figure 2). The data sets between 20 and 2 cGy $min^{-1}$ show very little dose-rate effect but at lower dose rates the data do diverge from the acute survival curve (shown by the dashed line). Reproducibility of the data at 0.25 cGy $min^{-1}$ was poor.

The data on HX142 are at five reduced dose rates (Figure 3). Again, there is little dose-rate effect for rates in excess of 1 cGy $min^{-1}$.

Each set of data has been fitted with the linear quadratic equation and the resulting parameters are shown in Table I. Comparison between the cell lines is facilitated by plotting isoeffect curves; Figure 4 shows the dose ($D_{0.01}$) required to produce a surviving fraction of 0.01 as a function of dose rate.

The data for each cell line have also been fitted using the incomplete repair model (Thames, 1985). Data at all dose

Correspondence: T.J. McMillan.
Received 23 March 1990; and in revised form 29 June 1990.

**Figure 1** Cell survival curves for HX138 and HX142 irradiated at high dose-rate (90 cGy $min^{-1}$).

**Table I** Parameters of linear-quadratic equation fitted to the survival curves

| *Cell line* | *Dose rate* (*cGy* $min^{-1}$) | α | β |
|---|---|---|---|
| HX138 | 90 | 1.23 ± 0.0006 | 0.0068 ± 0.0001 |
| | 20 | 0.65 ± 0.15 | 0.073 ± 0.030 |
| | 10 | 1.01 ± 0.22 | 0.045 ± 0.047 |
| | 5 | 0.78 ± 0.13 | 0.085 ± 0.029 |
| | 2 | 1.01 ± 0.033 | 0.0061 ± 0.016 |
| | 1 | 0.81 ± 0.006 | 0.0056 ± 0.022 |
| | 0.5 | 0.16 ± 0.26 | 0.094 ± 0.042 |
| | 0.25 | 0.98 ± 0.30 | − 0.051 ± 0.04 |
| HX142 | 90 | 0.92 ± 0.009 | 0.097 ± 0.008 |
| | 20 | 1.28 ± 0.17 | 0.0013 ± 0.032 |
| | 2 | 1.26 ± 0.0003 | − 0.006 ± 0.006 |
| | 1 | 0.66 ± 0.027 | 0.051 ± 0.004 |
| | 0.5 | 0.66 ± 0.06 | 0.076 ± 0.008 |
| | 0.25 | 1.03 ± 0.10 | − 0.03 ± 0.02 |

Units: α $Gy^{-1}$; β $Gy^{-2}$. Values are mean ± s.e.

rates were simultaneously fitted by minimising the mean square deviations of the experimental points from their respective calculated curve. The resulting parameters are given in Table II. $D_{0.01}$ values have also been calculated from the model and the values of these are shown as the full lines in Figure 4. It should be stressed that these lines are not fitted to the points shown in Figure 4 but to all the experimental data. Nevertheless, the agreement between model and data in each case is reasonably good.

*Split-dose recovery*

Split-dose experiments were performed on HX138 using an equal dose split (1 + 1, 2 + 2 Gy, etc.) at a range of dose levels from 1 to 6 Gy. Results were expressed as recovery ratio (RR), defined as the ratio of survival from the divided dose to that of the equivalent single dose. As a function of the time between doses, RR increased to reach a plateau level at about 90–120 min. The maximum recovery ratios ($RR_{max}$) were therefore taken as the mean of three values beyond 2 hours.

The linear-quadratic equation predicts that the $RR_{max}$ should increase rapidly with dose according to the relation $RR_{max} = \exp(2\beta d^2)$ (Thames, 1985; Steel *et al.*, 1987). The relationship between $RR_{max}$ and $2d^2$ is plotted in Figure 5 for HX138 and for HX142 (from Peacock *et al.*, 1988). As expected from the above equation the results are consistent with straight lines through the origin (Figure 5). The slope of these lines gives a value for β, which (because of its mode of derivation) we call $\beta_{RR}$. Values for $\beta_{RR}$ are given in Table II.

**Table II** Summary of survival curve parameters

| | *Acute survival curve* | | *Incomplete repair model* | | | *Split-dose recovery* |
|---|---|---|---|---|---|---|
| *Cell line* | α | β | α | β | $T_{\frac{1}{2}}$ | $\beta_{RR}$ |
| HX138 | 1.23 | 0.007 | 0.65 | 0.13 | 68 | 0.115 |
| HX142 | 0.92 | 0.097 | 0.90 | 0.096 | 73 | 0.059 |

Units: α $Gy^{-1}$; β $Gy^{-2}$; half-time in minutes.

### Discussion

The high radiosensitivity of cell lines derived from human neuroblastoma has been demonstrated in previous studies (Ohnuma *et al.*, 1977; Deacon *et al.*, 1985) although it was not seen in the work of Weichselbaum *et al.* (1980). For the cell lines that we have studied the survival curves observed at high dose rate are comparable to those demonstrated for transformed fibroblasts from ataxia-telangiectasia patients, a well established radiosensitive syndrome (McKinnon, 1987; McMillan *et al.*, 1989). In recent years it has been found that $SF_2$, the survival for a dose of 2 Gy, is a parameter of radiosensitivity that is not only clinically relevant but also shows good discrimination between cell lines (Deacon *et al.*, 1984; Fertil & Malaise, 1981). The $SF_2$ values for the two cell lines studied here are 0.083 and 0.11, which places them among the most radiosensitive human tumour cell lines so far reported.

On the basis of less complete data we previously concluded that the HX138 cell line showed no dose-rate effect (Deacon, 1987; Steel *et al.*, 1987). We have now examined the radiosensitivity of these cells at eight dose rates down to 0.25 Gy $min^{-1}$. As shown in Figures 2 and 4, there is evidence at the lower rates for a clear sparing effect. In the HX142 cell line our data at six dose rates demonstrate a smaller but distinct sparing effect below 2 cGy $min^{-1}$.

In order to quantitate the change in cell survival as the dose-rate is altered we have fitted the data with the incomplete repair model described by Thames (1985). This model is linear-quadratic in form at high dose rate and it incorporates a parameter which determines the speed with which the β-component of cell killing disappears after an increment of radiation exposure, and therefore the kinetics of the dose-rate effect. From this the half time for recovery, $T_{\frac{1}{2}}$, can be derived. It should be emphasised that we are making no conclusions regarding the mechanisms underlying the parameters in this model. Rather we are simply using it to describe the dose-rate effect in these cell lines.

Our data appear to be reasonably consistent with this model, although the data at some individual dose rates are

**Figure 2** Survival curves for HX138 after irradiation at dose-rates from 20 to 0.25 cGy min$^{-1}$. The dashed lines represent the acute survival curve (90 cGy min$^{-1}$).

**Figure 3** Survival curves for HX142 after irradiation at dose-rates from 20 to 0.25 cGy min$^{-1}$. The dashed lines represent the acute survival curve (90 cGy min$^{-1}$).

**Figure 4** Isoeffect curves for HX138 and HX142 as a function of dose rate. $D_{0.01}$ is the dose to reduce survival to 0.01. The full lines are calculated using the incomplete repair model, globally fitted to the cell survival data. Parameter values are given in Table II.

**Figure 5** A plot of log(RR) against $2d^2$ for split-dose recovery experiments on the two neuroblastomas. ●, HX138; □, HX142. Data for HX142 are taken from Peacock et al. (1988).

not well fitted. For instance, in HX138 at 20 cGy min$^{-1}$ (Figures 2 and 4) the data suggest a change in sensitivity that is not confirmed by the trend with dose rate. This analysis leads to the parameter values shown in Table II. The values for $\alpha$ and $\beta$ for HX142 correspond closely to those obtained by fitting a linear-quadratic equation to the acute survival data alone. For HX138, there are considerable differences, the value for $\alpha$ being half the acute survival value. These differences arise because the acute survival data define an almost straight survival curve (with $\beta$ correspondingly low) but the incomplete repair model requires a slight curvature in order to model the sparing effect that appears at low dose rates (in the model the slope of the low dose-rate curve must extrapolate the initial slope of the acute survival curve). Which of these two $\alpha$-values is the more reliable is not clear. The fit to the acute survival data is subject to problems because of the trade-off of one parameter in the equation against the other. On the other hand, the incomplete repair model separates the parameters more easily but it makes assumptions regarding the relationship between the shape of the acute survival curve and the rate and extent of the dose-rate effect.

The present studies have gone down to lower dose rates than we have previously used (Steel et al., 1987). The limiting factor is the need to avoid cell proliferation during radiation exposure. The population doubling times of untreated HX138 and HX142 cultures are 24–30 h. Irradiation will induce growth retardation, and we have therefore reasoned that an overall exposure time of up to 40 h would be unlikely to allow more than about one doubling of cell number. The time taken to deliver 6 Gy in these very radiosensitive cells was 10 h at 1 cGy min$^{-1}$ and 40 h at 0.25 cGy min$^{-1}$. We cannot rule out the possibility that there may have been a small effect of cell proliferation at the lowest dose rates; indeed this may be the reason why for both cell lines the points in Figure 4 at 0.25 cGy min$^{-1}$ lie above the theoretical curve and produce negative values for $\beta$ in the linear quadratic fit. If proliferation does occur, then the progression of cells through phases of the cell cycle with differing radiosensitivity may also alter the response at low dose-rate. The effect of this should be to increase sensitivity and therefore is not likely to increase the dose-rate effect.

Split-dose experiments at multiple dose levels provide an alternative measure of cellular recovery, as indicated by the $\beta$ parameter in the linear-quadratic equation (Peacock et al., 1988). Our data obtained in this way were consistent with the implications of the linear-quadratic equation in showing a linear increase of log (RR) with $2d^2$ (Figure 5). This does not, however, preclude an exponential portion of the curve at high doses. For HX138 cells the value for $\beta_{RR}$ thus found was close to that from the incomplete repair model (Table II). For HX142 we have previously shown that the values differ by a factor of 2 (Peacock et al., 1988). If this difference is real, a possible explanation might be sought in terms of inducible repair (Friedberg, 1985; Shadley & Wiencke, 1989). We doubt this, however, because in studies on other human tumour cell lines there does not appear to be a significant trend towards the two $\beta$ values differing in a consistent way.

The values of $\beta_{RR}$ obtained for these lines are higher than we have obtained for any of our more resistant human tumour cell lines (Peacock et al., 1988; Yang et al., 1990). This shows directly that for any given dose the recovery observed in a split-dose experiment is greatest in the more sensitive lines. These sensitive neuroblastoma cell lines are certainly not recovery deficient and therefore may not be deficient in repair of DNA lesions. In this regard the neuroblastoma cells differ from ataxia telangiectasia fibroblasts which, although they are similarly radiosensitive, show less recovery after irradiation than their normal counterpart (Cox, 1982; Peacock et al., 1989).

The results that we have obtained indicate the contrast between 'recovery capacity' (which we would identify with the value of $\beta$) and 'dose recovery' in a fractionation or low dose-rate treatment. Neuroblastoma cells have a high recovery capacity, but because they have high $\alpha$ values (and therefore steep survival curves both at high and low dose rate) we observe low values for dose recovery. This contrast has been discussed further in a recent review paper (Steel et al., 1989).

From a mechanistic molecular point of view these results have important implications. It has until recently been believed that the major determinant of cellular sensitivity was repair capacity and this was often inferred from studies of cellular recovery. We would therefore have expected to find obvious recovery deficiencies in the neuroblastoma cells. That this was not the case suggests that recovery and hence repair is not a major determinant of cellular radiosensitivity. Measurement of DNA double-strand breaks using neutral filter elution has suggested that the level of damage initially induced in DNA by ionising radiation may be an additional factor (Radford, 1986; McMillan et al., 1989, 1990).

We are grateful to Miss R. Couch and Mrs S. Stockbridge for preparing this manuscript. This work was funded by grants from the Cancer Research Campaign.

### References

COURTENAY, V.D. & MILLS, J. (1978). An *in vitro* colony assay for human tumours grown in immune-suppressed mice and treated *in vivo* with cytotoxic agents. *Br. J. Cancer*, **37**, 261.

COX, R. (1982). A cellular description of the repair defect in ataxia-telangiectasia. In *Ataxia-telangiectasia: a Cellular and Molecular Link between Cancer, Neuropathology and Immune Deficiency*, Bridges, B.A. & Harnden, D.G. (eds) p. 141. Wiley: Chichester.

DEACON, J. (1987). The radiobiology of neuroblastoma. MD Thesis. University of London.

DEACON, J., PECKHAM, M.J. & STEEL, G.G. (1984). The radioresponsiveness of human tumours and the initial slope of the cell survival curve. *Radiother. Oncol.*, **2**, 317.

DEACON, J.M., WILSON, P. & PECKHAM, M.J. (1985). The radiobiology of human neuroblastoma. *Radiother. Oncol.*, **3**, 210.

FERTIL, B. & MALAISE, E.P. (1981). Inherent cellular sensitivity as a basic concept for human tumour radiotherapy. *Int. J. Radiat. Oncol. Biol. Phys.*, **7**, 621.

FRIEDBERG, E.C. (1985). *DNA Repair*. WH Freeman: San Francisco.

JACOBSON, H.M., MARCUS, R.B., THAR, T.L., MILLION, R.R., GRAHAM-POLE, J.R. & TALBERTS, J.L. (1983). Pediatric neuroblastoma: postoperative radiation therapy using less than 2000 rad. *Int. J. Radiat. Oncol. Biol. Phys.*, **9**, 501.

MCKINNON, P.J. (1987). Ataxia telangiectasia: an inherited disorder of ionizing-radiation sensitivity in man. *Human Genet.*, **75**, 197.

MCMILLAN, T.J., CASSONI, A.M., EDWARDS, S., HOLMES, A. & PEACOCK, J.H. (1990). The relationship of DNA double strand break induction to radiosensitivity in human tumour cell lines. *Int. J. Radiat. Biol.* (in the press).

MCMILLAN, T.J., EADY, J.J., HOLMES, A., PEACOCK, J.H. & STEEL, G.G. (1989). The radiosensitivity of human neuroblastoma: a cellular and molecular study. *Int. J. Radiat. Biol.*, **56**, 651.

OHNUMA, N., KASUGA, T., NOJIRI, I. & FURUSE, T. (1977). Radiosensitivity of human neuroblastoma cell line (NB-1). *Gann*, **68**, 711.

PEACOCK, J.H., CASSONI, A.M., MCMILLAN, T.J. & STEEL, G.G. (1988). Radiosensitive human tumour cell lines may not be recovery deficient. *Int. J. Radiat. Biol.*, **54**, 945.

PEACOCK, J.H., EADY, J.J., EDWARDS, S., HOLMES, A., MCMILLAN, T.J. & STEEL, G.G. (1989). Initial damage or repair as the major determinant of cellular radiosensitivity. *Int. J. Radiat. Biol.*, **56**, 543.

PINKERTON, C.R. (1990). Where next with therapy in advanced neuroblastoma? *Br. J. Cancer*, **61**, 351.

RADFORD, I.R. (1986). Evidence for a general relationship between the induced level of DNA double strand breakage and cell killing after x-irradiation of mammalian cells. *Int. J. Radiat. Biol.*, **49**, 611.

SHADLEY, J.D. & WIENCKE, J.K. (1989). Induction of the adaptive response by X-rays is dependent on radiation intensity. *Int. J. Radiat. Biol.*, **56**, 107.

STEEL, G.G., DEACON, J.M., DUCHESNE, G.M., HORWICH, A., KELLAND, L.R. & PEACOCK, J.H. (1987). The dose-rate effect in human tumour cells. *Radiother. Oncol.*, **9**, 299.

STEEL, G.G., MCMILLAN, T.J. & PEACOCK, J.H. (1989). The picture has changed in the 1980s. *Int. J. Radiat. Biol.*, **56**, 525.

THAMES, H.D. (1985). An 'incomplete-repair' model for survival after fractionated and continuous irradiations. *Int. J. Radiat. Biol.*, **47**, 319.

WEICHSELBAUM, R.R., NOVE, J. & LITTLE, J.B. (1980). X-ray sensitivity of human tumour cells *in vitro*. *Int. J. Radiat. Oncol. Biol. Phys.*, **6**, 437.

YANG, X., DARLING, J.L., MCMILLAN, T.J., PEACOCK, J.H. & STEEL, G.G. (1990). Radiosensitivity, recovery and dose-rate effect in three human glioma cell lines. *Radiother. Oncol.* (in the press).